# Community-acquired fulminant *Clostridioides* (*Clostridium*) *difficile* infection by ribotype 027 isolate in Japan: a case report

**DOI:** 10.1186/s40792-021-01220-9

**Published:** 2021-06-08

**Authors:** Masayuki Hiraki, Rei Suzuki, Nobuo Tanaka, Hiroki Fukunaga, Yoshinori Kinoshita, Hayato Kimura, Shusaku Tsutsui, Masaru Murata, Shunji Morita

**Affiliations:** 1 Department of Surgery, Itami City Hospital, 1-100 Koyaike, Itami-shi, Hyogo 664-8540 Japan; 2 Department of Surgery, Kansai Rosai Hospital, Japan Organization of Occupational Health and Safety, 3-1-69 Inabaso, Amagasaki, Hyogo 660-8511 Japan; 3 Department of Respiratory Medicine, Itami City Hospital, 1-100 Koyaike, Itami-shi, Hyogo 664-8540 Japan; 4 Department of Diagnostic Pathology, Itami City Hospital, 1-100 Koyaike, Itami-shi, Hyogo 664-8540 Japan; 5 Department of Gastroenterology, Itami City Hospital, 1-100 Koyaike, Itami-shi, Hyogo 664-8540 Japan

**Keywords:** *Clostridioides* (*Clostridium*) *difficile* infection, Fulminant colitis, Proctocolectomy, PCR-ribotype 027, *Helicobacter pylori* eradication therapy, Community-acquired CDI

## Abstract

**Background:**

*Clostridioides* (*Clostridium*) *difficile* infection (CDI) has become an increasingly significant disease not only as healthcare-associated infection, but also as community-acquired (CA) infection worldwide. CDI caused by the NAP1/BI/027 strain is reported to be more severe, difficult to cure, and frequently associated with recurrences in North America and Europe.

**Case presentation:**

A 68-year-old woman was referred to our hospital for continuous lower abdominal pain 4 weeks after eradication therapy against *Helicobacter pylori*. While she was treated with fasting on the suspicion of ischemic colitis, she experienced septic shock. Emergent subtotal proctocolectomy revealed fulminant pseudomembranous *C. difficile* colitis. The *C. difficile* isolate recovered from the patient was identified as ribotype 027, which has been reported to be uncommon in Japan.

**Conclusion:**

We report a rare case of CA fulminant pseudomembranous colitis caused by ribotype 027 *C. difficile* after *H. pylori* eradication therapy.

## Background

*Clostridioides difficile* infection (CDI) is the leading cause of healthcare-associated infectious diarrhea and, therefore, there has been continued expanding interest in the epidemiology, prevention, diagnosis, and treatment of CDI [1]. Furthermore, in recent years, community-acquired CDI have been reported [2]. National surveillance has already been carried out abroad, and there are guidelines for the prevention of CDI. On the other hands, in Japan the actual state of CDI remains unknown since there is no national data. Kato et al. reported a high CDI incidence in Japanese hospitals because of the presence of a large population with numerous risk factors for CDI, such as advanced age, widespread use of broad-spectrum antibiotics, severe comorbidities, and long hospital stays and suggested a high CDI incidence as healthcare-associated infection in Japan [3]. However, to our knowledge, there are few reports on details of community-acquired (CA) CDI in Japan.

The symptoms of CDI are highly variable from asymptomatic carriage to severe diarrhea that can progress to toxic megacolon, fulminant colitis, and septic shock. Pseudomembranous colitis is one of the manifestations of severe CDI. Severe CDI occasionally requires surgical intervention, including hemicolectomy or subtotal colectomy, in cases of fulminant diseases [4]. The severity of CDI has been reported to increase with increasing incidence during outbreaks and emergence of the polymerase chain reaction (PCR) ribotype 027 epidemic strain (also known as the North American pulsed-field type 1 [NAP1] or restriction endonuclease analysis pattern “BI”) [5]. Although the ribotype 027 strain, known as a virulent strain, has caused outbreaks across North America, England, and parts of continental Europe, it has not been dominant in Asia [6]. We here report a rare case with ribotype 027 in Japan. She suffered from CA fulminant *C. difficile* colitis after *Helicobacter pylori* (*H. pylori*) eradication therapy and eventually required subtotal proctocolectomy.

## Case presentation

A 68-year-old woman received *H. pylori* eradication therapy in 2017 at a nearby medical clinic, with a potassium-competitive acid blocker (vonoprazan), amoxicillin, and clarithromycin. She had no history of other comorbidities or prior hospital admission. Four weeks after eradication therapy, she developed lower abdominal pain and revisited the clinic. A single dose of ceftriaxone was administered, but her symptoms did not improve.

**Fig. 1:**
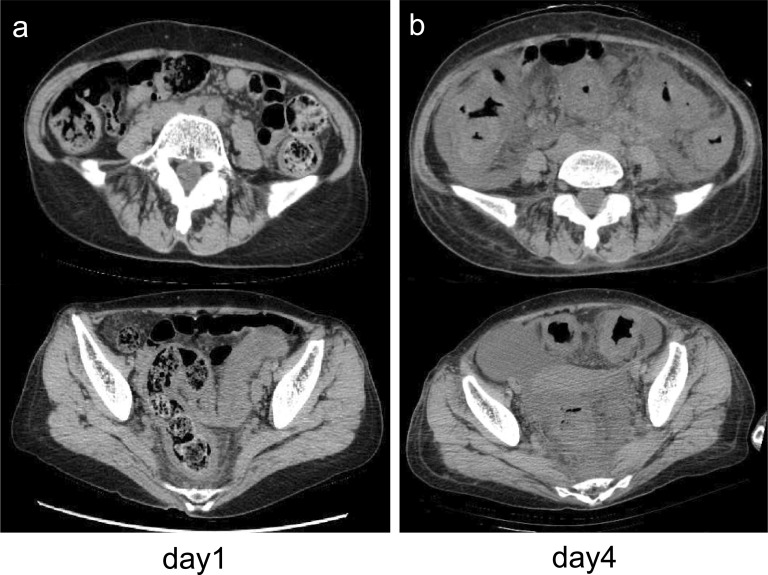
CT shows progressively worsening edematous changes in the intestinal wall and increased ascites from the first (**a**) to the fourth (**b**) hospital day

**Fig. 2:**
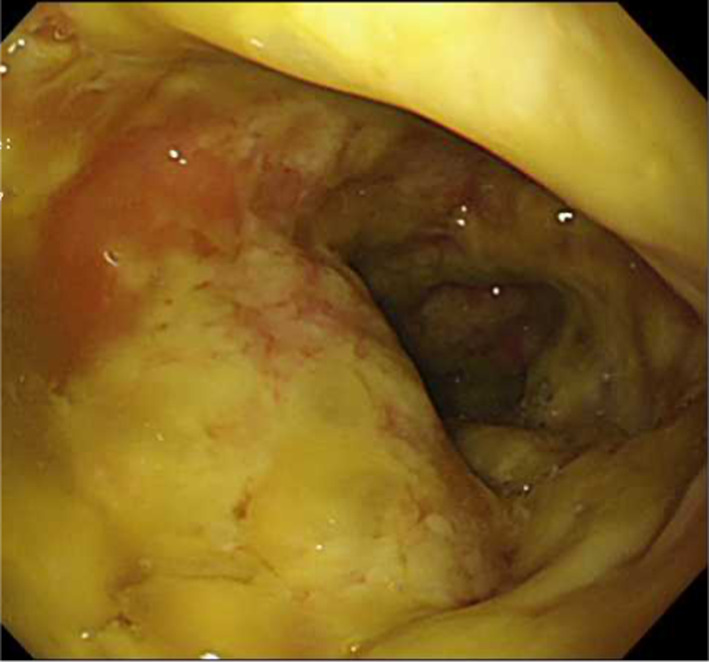
Urgent sigmoidoscopy shows widespread yellow-white purulent plaques on the surfaces of the sigmoid–rectal mucosa

**Fig. 3:**
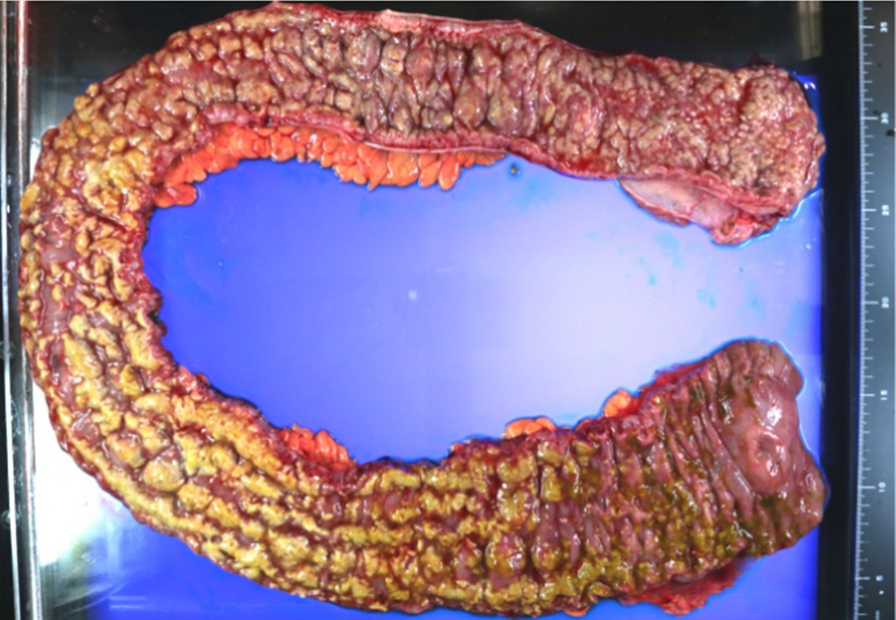
The resected specimen has the highly edematous mucosa. A yellowish-white pseudomembrane is widely formed in a patchy or cohesive form on the mucosa of the entire colon and rectum

**Fig. 4:**
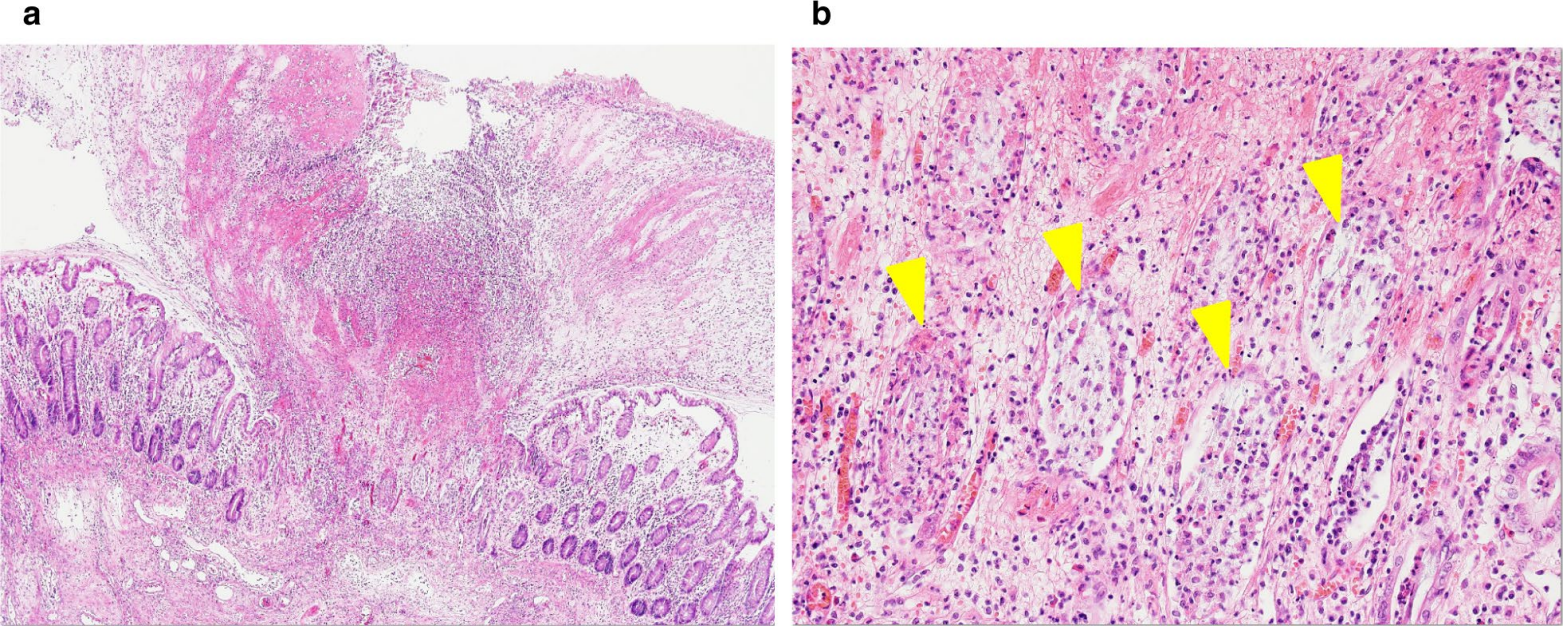
The microscopic findings. **a** There is a volcanic eruption image, in which the pseudomembrane component is ejected into the intestinal lumen at the mucosal defect. The pseudomembrane histologically consists of fibrin, mucus, neutrophils, and nuclear debris. **b** The expanded frame of the crypt remains on the mucosa of the pseudomembrane attachment site, and the dropped-out epithelial cells are seen in the lumen

**Fig. 5:**
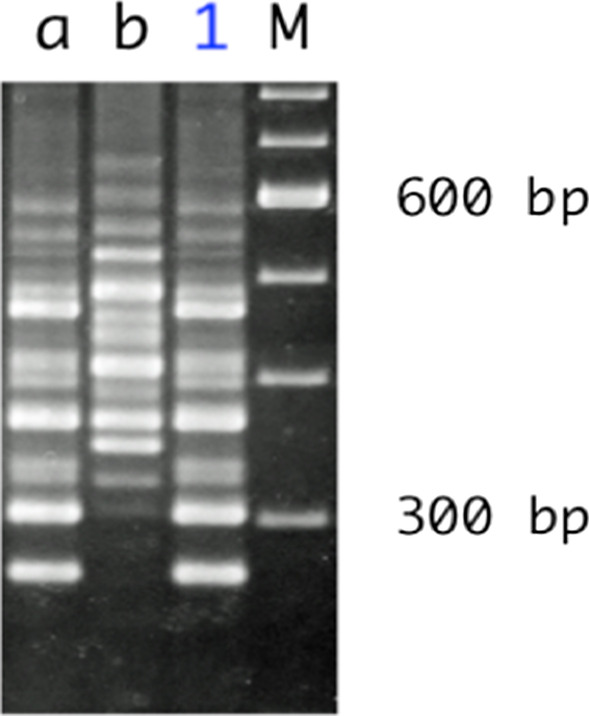
PCR-ribotype pattern of the isolate recovered from the present case. Lane M, 100-bp ladder as a DNA size marker; lane 1, the isolate from our case; lane a, ribotype 027; lane b, ribotype 078

She was referred to our hospital 3 days after the administration of ceftriaxone. Her blood pressure, temperature, and heart rate were 127/65 mmHg, 37.5 °C, and 88 beats/min, respectively. She had abdominal tenderness in the left lower quadrant without muscular defense and no diarrhea. Computed tomography (CT) showed edematous thickening of the intestinal wall from the descending colon to the rectum, with increased densities of the surrounding fat tissues (Fig. 1a). Moreover, the laboratory results were as follows: white blood cell (WBC) count, 12,200/μL; hemoglobin, 11.2 g/dL; platelet (PLT) count, 18.3 × 10^4^/μL; C-reactive protein (CRP), 2.32 mg/dL; T-bil, 0.77 IU/L; AST, 17 IU/L; ALT, 11 IU/L; BUN, 10.6 mg/dL; creatinine, 0.49 mg/dL; albumin, 3.3 g/dL; FDP, 4.0 μg/dL; and prothrombin time (PT), 13.4 s (PT-INR, 1.13). She began to have light pink mucous stool immediately after the visit and was admitted to our hospital on the suspicion of ischemic colitis. She was followed conservatively with fasting, but the abdominal pain persisted with watery diarrhea. On the third day of hospitalization, she experienced septic shock with a systolic blood pressure of 60 mmHg and temperature of 38.6 °C. The WBC count and hemoglobin and CRP levels were 26,800/μL, 11.0 g/dL, and 19.3 mg/dL, respectively. She was transferred to the intensive care unit and administered meropenem on the suspicion of bacterial enteritis. On the fourth day, the inflammatory response further worsened with a WBC count of 34,400/μL and CRP of 22.8 mg/dL. Other laboratory results were as follows: hemoglobin, 11.2 g/dL; PLT count, 20.3 × 10^4^/μL; T-bil, 0.29 IU/L; AST, 13 IU/L; ALT, 9 IU/L; BUN, 9.7 mg/dL; creatinine, 0.51 mg/dL; albumin, 1.5 g/dL; FDP, 6.6 μg/dL; fibrinogen quantity, 389 mg/dL; and PT, 15.5 s (PT-INR, 1.31). Disseminated intravascular coagulation (DIC) score was 2 according to criterion of the Scientific Subcommittee on DIC of the International Society for Thrombosis and Haemostasis (ISTH). CT was repeated and demonstrated the extended wall thickening throughout the entire colon and increased ascites (Fig. 1b). Vancomycin was additionally administered intravenously as empirical treatment on the suspicion of severe enteritis. Moreover, metronidazole was intravenously administered considering the possibility of CD colitis. At this point, the physician consulted with the surgeon about the treatment strategy. Emergent sigmoidoscopy showed yellow-white purulent plaques on the mucosal surfaces of the rectum (Fig. 2). Soon after the endoscopic examination, she was intubated due to exacerbated circulatory and respiratory conditions. As colitis was judged to be severe, we decided to perform an emergency surgery. Surgical findings showed that much ascites was found in the abdomen and that the entire colon was markedly edematous and dilated with no obvious necrotic changes. Subtotal proctocolectomy from the cecum to the lower rectum was performed with ileostomy formation. After a drain tube was inserted at the pelvic bottom, the abdomen was closed. Perioperative outcomes were as follows: operation time, 250 min; loss of bleeding, 200 mL; complications, none; and length of hospital stay after surgery, 22 days. Severe pseudomembranous changes were observed on the mucosal surface of the entire colon and rectum in the resected specimen (Fig. 3). The diagnosis of pseudomembranous colitis was also supported by the typical microscopic appearances (Fig. 4). She was additionally treated with intravenous metronidazole administration (1500 mg/day) until 10 days after surgery and discharged 22 days after surgery. The enzyme immunoassay tests of the stool specimen obtained on the third day were negative for toxin A/B but positive for *C. difficile* glutamate dehydrogenase. It indicated that *C. difficile* was present but CD toxin was not detected. There were two possibilities: a non-CD toxin-producing strain or a false-negative strain due to the low sensitivity of the CD toxin test, which is actually a CD toxin-producing. Culture on the specimen with high sensitivity and specificity yielded *C. difficile*, which was toxin A-positive, toxin B-positive, and binary toxin-positive. The isolate was further identified as ribotype 027 (Fig. 5).

## Discussion

CDIs are the leading cause of healthcare-associated diarrhea worldwide and surpass all other healthcare-associated infections in some countries [5, 6]. The incidence rates of CDI dramatically increased early in the 2000s with the emergence of the epidemic ribotype 027 strain of *C. difficile*. In the United States, the CDI incidence increased from 4.5/1000 adult discharges in 2001 to 8.2/1000 discharges in 2010 [7]. The death numbers per million population in the United States also increased from 8.2 in 2001 to 23.7 in 2004 [8]. After the peak of CDI before 2010, the rates of CDI have declined remarkably in England and other parts of Europe, and those in the United States have plateaued [1].

In contrast to the prevalence of ribotype 027 in North America and Europe, there have been fewer reports of CDI by ribotype 027 in Asia, where ribotypes 001, 002, 014, 017, and 018 are prevalent [9]. In Japan, the first academic reports of ribotype 027 CDI were described in 2007 [10, 11], which were followed by a few sporadic reports [12]. There have been no reports on its outbreak in Japan. The actual prevalence of ribotype 027 in Japan is difficult to determine, as ribotyping has rarely been performed clinically. Mori et al. investigated 975 stool culture samples from inpatients in a university hospital in Japan, in which 177 *C. difficile* isolates were recovered and 127 isolates were toxigenic and 12 were positive for the binary toxin gene. However, clinically important ribotypes such as 027 and 078 were not identified [13]. Ribotyping is very crucial for understanding the epidemiology in Japan. However, it remains unknown whether there are clinical benefits in the treatment of each case since there have been few reports of ribotype 027.

Our case may be quite rare in terms of CA infection as well as disease severity and clinical settings. Severe complications of CDI, such as megacolon, perforation, colectomy, shock requiring vasopressor therapy, and death, were reported to be associated with an age of at least 65 years, nosocomial infection, high peripheral leukocyte count, high creatinine level, initial antibiotic treatment, immunosuppression, and tube feeding [14]. Reported colectomy rates in hospitalized patients with CDI range from 0.3 to 1.3% during endemic periods and 1.8% to 6.2% during epidemic periods [15]. It is uncertain whether *C. difficile* ribotype 027 in Asia has similar virulence to that in North America or Europe. In the whole genomic analysis, two ribotype 027 strains isolated from two Chinese patients with CDI were outside of two distinct epidemic lineages of *C. difficile* ribotype 027 that emerged in North America, suggesting different features among those strains [16]. Cheng et al. summarized 19 cases of ribotype 027 in Asia, which included only two cases of fulminant CDI [17].

Although CDI is classically considered a hospital-acquired infection, CA CDI has become an increasing public health threat. Previous reports suggested that substantial fractions of CDI cases were acquired in the community [5, 18, 19]. It was reported that 40% of patients with CA CDI required hospitalization, 20% had severe infection, 4.4% had severe complications, 20% ended up with treatment failure, and 28% had recurrent CDI [20]. The sources of and risk factors for CA CDI have not been well defined [1]. An analysis of 984 patients with CA CDI during 2009–2011 showed that a substantial proportion of patients did not have possible risk factors such as antibiotic exposure (18%) and proton pump inhibitors (31%) [21]. Moreover, related academic societies have reported that *H. pylori* triple-therapy is associated with CDI with an incidence of approximately 1% [22]. Our case had the risk of antibiotic exposure, the most important modifiable risk factor for CDI development [1, 22] 4 weeks before onset. The duration between drug administration and CDI onset seems to be reasonable, as the highest risk of CDI (seven- to tenfold increase) was observed during antibiotic therapy and in the first month after cessation of the therapy [23]. Another possible risk factor in our case was the use of stomach-acid suppressants although its role in CDI remains controversial [1, 19].

Emergency surgery is often required for fulminant CD colitis. The reasons are refractory cases, massive bleeding, paralytic ileus, sepsis, multiple organ failure, toxic megacolon and intestinal perforation [24]. Early surgical intervention is required in treatment-refractory cases because these complications increase the fatality rate. Lee et al. examined predictors of mortality after emergency colectomy for CD colitis and listed age > 80, preoperative septic shock, preoperative severe COPD, preoperative dialysis dependence, postoperative cardiac arrest, intraoperative wound infection, PLT count (× 10^3^/mm^3^) < 150, INR > 2, and BUN (mg/dL) > 40 as risk factors [25]. Therefore, our case was at high risk of mortality because she was in septic shock before surgery. According to the Infectious Diseases Society of America (IDSA) guidelines, the first-line treatment for fulminant CD enteritis is vancomycin administered orally. Moreover, if the patients become severe, subtotal colectomy with preservation of the rectum is strongly recommended [26]. Indeed, it is very important to diagnose and treat CD enteritis at an early stage, but If the condition becomes severe, immediate surgical intervention should not be hesitated.

## Conclusions

We unexpectedly had a fulminant CA CDI by ribotype 027. Although there have been few reports on the epidemiology of ribotype 027 in Japan, binary toxin-positive subtype, including ribotype 027 strain, may be associated with aggravation.

Since antibiotic drugs are frequently prescribed for various medical situations including *H. pylori* eradication therapy, we should be aware of the risk of CDI which could lead to a severe condition even in outpatient settings.

## Abbreviations

CDI: *Clostridioides difficile* infection
*H*. *pylori*: *Helicobacter pylori*
PCR: Polymerase chain reaction
CA: Community-acquired
CT: Computed tomography
WBC: White blood cell
CRP: C-reactive protein
PLT: Platelet
PT: Prothrombin time
DIC: Disseminated intravascular coagulation
